# Draft Genome Sequence of *Bacillus pseudalcaliphilus* PN-137^T^ (DSM 8725), an Alkaliphilic Halotolerant Bacterium Isolated from Garden Soils

**DOI:** 10.1128/genomeA.00919-15

**Published:** 2015-08-13

**Authors:** Jie-ping Wang, Bo Liu, Guo-hong Liu, Rong-feng Xiao, Xue-fang Zheng, Huai Shi, Ci-bin Ge

**Affiliations:** Agricultural Bio-Resources Research Institute, Fujian Academy of Agricultural Sciences, Fujian, China

## Abstract

*Bacillus pseudalcaliphilus* PN-137^T^ (DSM 8725) is a Gram-positive, spore-forming, alkaliphilic, and halotolerant bacterium. Here, we report the 4.49-Mb genome sequence of *B. pseudalcaliphilus* PN-137^T^, which will accelerate the application of this alkaliphile and provide useful information for genomic taxonomy and phylogenomics of *Bacillus*-like bacteria.

## GENOME ANNOUNCEMENT

*Bacillus pseudalcaliphilus* was described and named by Nielsen et al. in 1995 (1). Its type strain is PN-137 (= ATCC 700166 = CIP 105304 = DSM 8725 = LMG 17951). The strains of *B. pseudalcaliphilus* grow best at pH 10.0 but show no growth at pH 7.0, and they tolerate up to 10% NaC1, suggesting that this bacterium is alkaliphilic and halotolerant (1). Its phylogenetically close neighbors are the alkaliphilic species *Bacillus alcalophilus* (2), *Bacillus krulwichiae* (3), and *Bacillus trypoxylicola* (4), according to their 16S rRNA gene sequence similarity. Importantly, an alkaliphilic halotolerant strain, *B. pseudalcaliphilus* 8SB, can produce a new cyclodextrin glucanotransferase (CGTase, EC 2.4.1.19) with γ-cyclizing activity (5). It is of great application significance that the CGTase from *B. pseudalcaliphilus* is thermostable with a wide working pH range (from 5.0 to 11.0) and can convert a raw corn starch into only two types of cyclodextrins (CDs), β- and γ-CD (5). Moreover, γ-CD has wider applications in many industries (especially in the food and pharmaceutical industries) due to its larger internal cavity, higher water solubility, and more bioavailability than those of α- and β-CD (6).

Given the physiological properties, application prospects, and no available genomic information of *B. pseudalcaliphilus*, its type strain PN-137^T^ was selected as one of the research objects in our “genome sequencing project for genomic taxonomy and phylogenomics of *Bacillus*-like bacteria.” Here, we present the high-quality draft genome sequence of *B. pseudalcaliphilus* PN-137^T^ (DSM 8725).

The genome sequencing of *B. pseudalcaliphilus* PN-137^T^ was performed via the Illumina Hiseq 2500 system. Two DNA libraries with insert sizes of 500 and 5,000 bp were constructed and sequenced using the 2- × 150-bp paired-end sequencing strategy. After filtering of the 1.23 GMb raw data, 1.01 GMb clean data were obtained, providing approximately 200-fold coverage. The reads were assembled via SOAPdenovo software version 1.05 (7), using a key parameter K setting at 71. Through the data assembly, 25 scaffolds with total length 4,487,412 bp were obtained, and the scaffold *N*_50_ was 586,609 bp. The average length of the scaffolds was 179,496 bp, and the longest and shortest scaffolds were 1,421,391 bp and 542 bp, respectively. A total of 72.22% clean reads were aligned back to the genome, which covered 99.46% of the sequence.

The annotation of the genome was performed using the NCBI Prokaryotic Genomes Automatic Annotation Pipeline (PGAAP) (http://www.ncbi.nlm.nih.gov/genomes/static/Pipeline.html) utilizing GeneMark, Glimmer, and tRNAscan-SE tools (8). A total of 4,253 genes were predicted, including 4,019 coding sequences (CDS), 156 pseudo genes, 74 tRNAs, and 3 rRNA genes. Two clustered regularly interspaced short palindromic repeat (CRISPR) arrays were found in the draft genome. There were 2,935 and 2,005 genes assigned to COG and KEGG database, respectively. The average DNA G+C content was 37.93%, with a slight difference from the value of 38.2 to 39.0 mol% acquired by HPLC determination (1).

### Nucleotide sequence accession numbers.

This whole-genome shotgun project has been deposited at DDBJ/EMBL/GenBank under the accession no. LFJO00000000. The version described in this paper is version LFJO01000000.
